# Effectiveness of miltefosine in cutaneous leishmaniasis caused by *Leishmania tropica* in Pakistan after antimonial treatment failure or contraindications to first line therapy—A retrospective analysis

**DOI:** 10.1371/journal.pntd.0008988

**Published:** 2021-01-28

**Authors:** Suzette Kämink, Boota Masih, Noor Ali, Aman Ullah, Syed Juma Khan, Shakil Ashraf, Tetyana Pylypenko, Martin P. Grobusch, Jena Fernhout, Margriet den Boer, Koert Ritmeijer

**Affiliations:** 1 Médecins Sans Frontières, Quetta, Pakistan; 2 Center of Tropical Medicine and Travel Medicine, Department of Infectious Diseases, Amsterdam University Medical Centers, location AMC, Amsterdam Public Health, Amsterdam Infection and Immunity, University of Amsterdam, Amsterdam, The Netherlands; 3 Mohtarma Shaheed Benazir Bhutto General Hospital,Quetta, Pakistan; 4 Médecins Sans Frontières, Islamabad, Pakistan; 5 Médecins Sans Frontières, Amsterdam, the Netherlands; 6 Médecins Sans Frontières, London, United Kingdom; Universidade do Estado do Rio de Janeiro, BRAZIL

## Abstract

**Background:**

Cutaneous leishmaniasis (CL) is a neglected tropical skin disease, caused by *Leishmania* protozoa. In Pakistan, where CL caused by *L*. *tropica* is highly endemic, therapy with pentavalent antimonials is the standard of care, but has significant toxicity when used in systemic therapy, while are no evidence-based safer alternative treatment options for *L*. *tropica*. The efficacy of oral miltefosine has not been studied in CL caused by *L*. *tropica*. We evaluated effectiveness and tolerability of miltefosine in patients with previous treatment failure or with contraindications to systemic antimonial treatment.

**Methods:**

A retrospective review was conducted of a cohort of CL patients who were treated with a 28-day course of miltefosine between December 2017 and August 2019, in urban Quetta, Pakistan, an area endemic for *L*. *tropica*. Descriptive analyses were performed, and effectiveness was assessed by initial response after treatment, and final cure at routine follow up visits, six weeks to three months post-treatment. Tolerability was assessed by routinely reported adverse events.

**Results:**

Of the 76 CL patients in the cohort, 42 (55%) had contraindications to systemic antimonial treatment, and 34 (45%) had failure or relapse after antimonial treatment. Twelve patients defaulted during treatment and 12 patients were lost to follow up. In the remaining 52 patients, final cure rate was 77% (40/52). In those with contraindications to systemic antimonial treatment the final cure rate was 83% (24/29) and in the failure and relapse group 70% (16/23). Twenty-eight patients (40.0%) reported 39 mild to moderate adverse events with the main complaints being nausea (41.0%), general malaise (25.6%), and stomach pain (12.8%).

**Conclusion:**

Results indicate that miltefosine is an effective second line treatment in CL in areas endemic for *L*. *tropica*. Prospective studies with systematic follow up are needed to obtain definitive evidence of effectiveness and tolerability, including identification of risk factors for miltefosine treatment failure.

## Author summary

Cutaneous Leishmaniasis (CL) is a neglected tropical skin disease, which globally affects an estimated 0.6 to 1 million people. The skin disease is caused by the protozoa *Leishmania* and is transmitted by phlebotomine sandflies in Old World CL (OWCL). In Pakistan, CL is highly endemic, especially in the provinces Balochistan and Khyber Pashtunkwa, where *L*. *tropica* is the main causative species. In this context, untreated CL often leads to stigmatisation and severe (psycho)social suffering, due to the disfiguring ulcerating wounds and scars. The mainstay treatment is with pentavalent antimonials, and evidence for efficacy of alternative treatments for *L*. *tropica* is lacking. Médecins sans Frontières (MSF) has specialised treatment centres in Balochistan (Quetta and Kuchlak), where in December 2017 miltefosine was introduced for patients who did not respond to, or had contraindications for antimonials. In this study, we showed favourable outcomes of miltefosine as second-line treatment for these CL patients.

## Introduction

Cutaneous leishmaniasis is a parasitic neglected tropical disease, with an estimated annual incidence of 0.6–1.0 million new cases. The majority (76%) of cases occur in seven countries, which each reported more than 10,000 cases: Syria, Afghanistan, Pakistan, Iraq, Iran, Brazil and Algeria [1]. In Pakistan, more than 19,000 cases were reported in 2018; however, it is estimated that the true annual CL incidence in Pakistan might range between 50,000 and 100,000 cases [1]. Despite being a notifiable disease since 2017, it is likely underreported due to the non-availability of treatment in many parts of the country [1–3].

The four causative species in OWCL are *Leishmania tropica*, *L*. *major*, *L*. *infantum*, *and L*. *aethiopica* [4,5]. In Pakistan, *L*. *tropica* is the predominant species in the provinces Balochistan and Khyber Pakhtunkhwa and *L*. *major* in Punjab and Sindh [3,6–9]. *L*. *major* is spread zoonotically via mammal reservoirs, such as gerbils and jirds, through the bite of the female phlebotomine sandflies *Phlebotomus papatasi* or *P*. *salehi*. *L*. *tropica* is solely anthroponotically transmitted by the sandfly *P*. *sergenti* [10,11]. At the site of the sandfly bite, a papule or nodule appears, which may eventually develop into large plaques or ulcerating lesions, depending on *Leishmania* species and immune response of the host. The lesions may be self-healing; however, this can take several months to years, depending on the species. *L*. *major* lesions usually heal within two to eight months, while *L*. *tropica* lesions can take up to a year or longer, and without treatment can develop into chronic disfiguring lesions or swelling of the affected limb, foot or hand, causing disability and disfigurement [12]. The sandflies mainly bite on exposed body parts: face, legs, feet, arms and hands, and therefore these CL lesions (nodules, ulcers and/or plaques) and scars often lead to (psycho-) social problems, stigmatisation and discrimination in family and community, especially for young women [8,13,14].

For decades, the mainstay treatment of CL has been with pentavalent antimonials; meglumine antimoniate or sodium stibogluconate, with efficacy rates differing per species. The efficacy of antimonial treatment in *L*. *tropica* varies widely between studies, with reported cure rates between 44.8% and 96% [15–20]. A proportion of patients does not respond to standard antimonial treatment regimens, or relapses following treatment, despite several treatment courses applied via intralesional and/or intramuscular treatment routes. Antimonials may have serious toxic adverse effects if given systemically (via intramuscular or parenteral treatment routes); these comprise cardiac-, hepatic-, renal-, and pancreatic toxicities. This is a particular problem in elderly patients who often have underlying comorbidities, which may constitute a contraindication to systemic antimonial treatment. In these high-risk patient groups, the risks may outweigh the benefits of treating this non-fatal dermal disease. In pregnancy, antimonials may cause miscarriage and premature delivery. For this reason, systemic antimonial treatment is contraindicated in pregnancy [4].

Miltefosine is licensed for treatment of visceral leishmaniasis in South Asia and several forms of cutaneous and muco-cutaneous forms of leishmaniasis in South America. There is considerable evidence in the literature of the efficacy of oral miltefosine in treatment of CL caused by *L*. *major* [21–23]. Miltefosine is registered in Pakistan for use in leishmaniasis (produced and sold under the name Fosine). However, until now, only limited case studies have been conducted to evaluate the efficacy of miltefosine in CL caused by *L*. *tropica* which is generally considered to be less drug sensitive [24–26]. It is not (yet) included in the Pakistan national guidelines, although it is recommended by the WHO in the manual for case management of CL in the EMRO region (2014) as a second line treatment [27,28].

Treatment with oral miltefosine could have major benefits for CL patients, especially as second-line treatment for patients whose lesion(s) do not respond to the standard antimonial treatment, or for whom systemic antimony treatment is contraindicated (e.g. patients with cardiac, hepatic, renal or diabetic disease). However, an important drawback is the possible teratogenicity of miltefosine, which renders it contraindicated in pregnant women [29]. Non-pregnant women of reproductive age should receive effective (injectable) contraception during and until five months after treatment, which in practice is difficult to realize. In clinical studies conducted with miltefosine, the most common adverse events reported are gastrointestinal disturbances, such as nausea, vomiting (25–42% of the patients), diarrhoea (5–20%) and abdominal pain, all mostly mild/moderate, and transient. Headache (27–28%), drowsiness and general discomfort are also frequently reported [30–33].

In Pakistan, Médecins Sans Frontières (MSF) runs three clinics in urban Quetta (Balochistan Province), in an *L*.*tropica* endemic area. In 2017, for a study, species identification by PCR was conducted in one of these clinics; which revealed that 100% of the patient samples were identified as *L*. *tropica* [27]. Patients with CL are routinely treated with meglumine antimoniate, either by daily intramuscular (IM) injections of 20 mg/kg for 20 days, or by intralesional (IL) injections twice a week, for eight to 12 sessions with a dose depending on the number and size of the lesions [27,28].

We witnessed a steady increase in the number of elderly patients presenting with CL, as well as patients whose lesions did not respond to, or relapsed, after at least two courses of antimonial treatment, of which at least one course administered via the systemic route. In December 2017, MSF introduced miltefosine (IMPAVIDO, Knight Therapeutics Inc., USA) to treat those patients with contraindications to systemic antimonial treatment, or those patients whose lesions had not responded to, or relapsed after minimal two courses of antimonial treatment, of which at least one course via systemic treatment route. those This was based on the assumption that miltefosine would have significant efficacy in *L*. *tropica*, based on limited published case studies and its good efficacy in CL caused by *L*. *major*. The aim of this study was to evaluate the effectiveness and tolerability of miltefosine in CL patients from a *L*. *tropica* endemic area.

## Methods

### Ethics statement

This research fulfilled the exemption criteria set by the MSF Ethical Review Board (ERB) for a posteriori analyses of routinely collected clinical data, and thus did not require MSF ERB review [34]. It was conducted with permission from the Medical Director of the MSF Operational Centre Amsterdam. Studies are exempted from local ethics review in Pakistan if they do not require direct human participation and use already collected data for routine public health reasons [35].

### Study design and data source

A retrospective analysis was conducted on data collected of a clinical cohort of 76 patients with CL who had not responded to, or relapsed after at least two previous courses of antimonial treatment (of which one administered via the systemic route), or for whom systemic antimonial treatment was contraindicated. These patients attended MSF’s health centre in Kuchlak, or the CL clinics in Mohtarma Shaheed Benazir Bhutto General Hospital Quetta (MSBBGHQ) and Bolan Medical Complex Hospital (BMCH), both MoH hospitals supported by MSF in Quetta, Balochistan. The data were routinely collected as part of the medical programme between December 2017 and August 2019.

### Diagnosis of CL

All patients were clinically screened by a dermatologist. Typical combinations of signs and symptoms of skin lesions are considered as suspect for CL, such as raised edges or nodules on exposed parts of the body, not itchy and not painful, and with a duration of more than three weeks. All patients with suspected CL underwent further laboratory diagnostic evaluation. From a tissue specimen taken from the lesion(s) by a fine needle aspirate (FNA), a thin smear was prepared [36]. This smear was Giemsa stained and examined under a light microscopy; if *Leishmania* amastigotes were observed in 1000 microscopic fields by 1000 magnification, a sample was considered positive (graded from 1+ to 6+) and confirmed as CL [37]. In a minority of patients, no parasites were found in the smear. In these cases, a clinical diagnosis of CL by the dermatologist was decisive, after excluding differential diagnoses [37,38]. Based on results of molecular species differentiation in the study on the same patient population conducted in 2017, it was assumed that lesions were exclusively due to *L*. *tropica* [31].

### Treatment

Patients were treated with oral miltefosine for 28 days. The dosage for patients weighing 45 kg or more was 150mg/day (50 mg capsules three times per day), and patients between 30 kg and 45 kg received 100mg/day (50 mg capsules twice a day). For patients weighing less than 30 kg, an allometric dosing scheme by sex, weight and height defined the dose to be administered [21,39,40]. Miltefosine was provided to patients for one week at a time, divided in daily dosages. Patients were instructed to take the capsules 15–30 minutes after a meal to minimise gastrointestinal adverse events. Each week the patients had to come to the clinic for a refill, and to bring the empty blisters. These empty blisters were counted and checked to ensure the patients had taken the miltefosine capsules correctly and had been compliant with the prescribed therapy. The patients were asked to come back twice after discharge, with the final follow up six weeks after treatment (day 70). If the patient was female and in child bearing age (15–45 years old) she and her husband (if married) were told about the possible teratogenicity of the miltefosine and it was explained that there is a risk for the unborn child if she would become pregnant. Thus, to prevent her from getting pregnant from the start of the miltefosine therapy until five months after treatment, they needed to consent to accepting contraception with (injectable) medroxyprogesterone (Depo-Provera).

### Outcome parameters

The primary outcome was response to miltefosine measured during the final follow up visit. We conducted an analysis with the final outcomes documented as ‘final cure’, ‘relapse’ or ‘treatment failure’. All outcome definitions are in line with the CL treatment protocol: ‘Final cure’ was defined as 100% re-epithelisation (of a treated ulcer), or 100% flattening (of a treated non-ulcerated lesion) and absence of super-infections, three months after starting the treatment. ‘Treatment failure’ was defined as 1) if at the first follow up visit, three weeks after completing the treatment, an increase of the nodule, plaque or ulceration was observed; or 2) less than 50% was re-epithelialised (of a treated ulcer) or flattened (of an treated non-ulcerated lesion) six weeks after treatment or 3) if three months after the start of the treatment the lesion was not 100% re-epithelialised and no complete flattening was observed; or 4) if persistent signs of inflammation were observed at three months after the start of the treatment [28,41,42]. ‘Relapse’ was defined as if after complete initial epithelisation or flattening of a treated ulcer or non-ulcerated treated lesion CL re-appeared with active and raised edges, with or without extension to further locations [28]. The secondary outcome, initial treatment response, was documented at the end of the treatment, when the patient was discharged: ‘positive initial treatment response’ was defined as substantial clinical healing by visual observation: start of re-epithelizing of ulcers and/or flattening of raised edges of the lesion at the end of the treatment, whereas ‘initial treatment failure’ was defined as no clinical response being observed or an experienced CL staff/dermatologist had doubt that the healing process had begun; and/or patients themselves reported that no effect of the treatment was seen at the end of the treatment. A ‘default’ patient was defined as an individual who dropped out during treatment, and did not complete the course of 28 days of miltefosine. A ‘Lost-To-Follow-Up’ (LTFU) patient was defined as a patient who completed the treatment but did not come for any of the follow-up visits and who we were unable to trace. The follow-up period was defined according the MSF’s guidelines for case management of CL in the routine CL programme.

### Adverse event analysis

The experienced nursing staff are well trained and experienced in the management of CL patients, and take special care of their wellbeing, which includes monitoring and recording the effects and adverse events of drugs, and monitoring the mental health of the patients. This is the routine procedure in the management of all CL patients in the MSF treatment programme. During treatment and at each follow up visit, the nursing staff ask about the general health, experiences and complaints of the patients and provide counselling and coaching. Adverse events which the patient reported were recorded by the nursing staff, and graded afterwards during data analysis by the researchers as ‘mild’, ‘moderate’ and ‘severe’ adverse event (AE) or serious adverse event (SAE). We defined adverse events that did not lead to treatment interruption as ‘mild’, and adverse events that led to default from treatment or treatment interruption as ‘moderate’. In case the AE led to hospitalization and discontinuation of the miltefosine treatment, it was defined as ‘severe’. Serious AEs were defined if life-threatening. The AEs were entered in the electronic and physical line list by the nursing staff and analysed for this study. More than one AE could be reported per patient.

### Statistical analysis

#### Data and statistical analysis

Key variables included dates of treatment and follow-up visits, sociodemographic, diagnostic and clinical characteristics of the CL lesions, treatment regime, initial treatment outcome and follow-up outcomes. Only patients who completed the treatment were included, whereas those who defaulted were excluded from analysis. Descriptive statistics were used to report sex, age, type of lesion, location and duration of lesion, number of lesions and reason to treat with miltefosine. Categorical (dichotomous and nominal) and continuous variables of the anthropometrical measurements and demographic characteristics were summarised using number of patients (n) and percentage (%)and/or median and interquartile range (IQR).

Data were stratified into two groups; 1) patients with a contraindication for systemic antimonial treatment due to an underlying cardiac disease, ECG abnormalities, renal/hepatic disease, diabetes, or any other condition that is a contraindication for pentavalent antimonials; 2) patients who had failed to cure or relapsed after at least two treatment courses of meglumine antimoniate, of which at least one course was administered via the systemic route.

We analysed stratified and unstratified data. The groups were compared with the odds ratio as effect size, but the cohort was too small to conduct further logistic analyses.

For statistical analysis, STATA 15.1 was used. Copyright 1985–2017 StataCorp LLC, Statistics/Data Analysis StataCorp, Texas, USA, http://www.stata.com

## Results

Between December 2017 and August 2019, 76 patients with CL were treated with miltefosine.

### Diagnosis

The diagnosis of CL was established by physical examination by a dermatologist and parasitological confirmation in a FNA smear. Of these 76 patients, 54 (71.1%) patients screened positive for CL by this laboratory test and 22 (28.9%) patients were clinically diagnosed by the experienced dermatologist, with a negative CL test.

### Patient characteristics

Of the 76 patients, 45 (59.2%) were male. The median age was 48 years old (IQR 13–59). The mean number of lesions per patients was 2.5, while four (5.3%) patients had more than eight lesions. The median duration of the lesions, the period between onset of lesions and start of miltefosine, was seven months (IQR 5–12). The majority of lesions, 56.6%, were plaques, while 19.7% were nodules and 23.7% ulcers.

The 76 patients together had 192 lesions; 61 (80.3%) patients had lesions on the face, of whom 11 also had lesions elsewhere on the body (upper or lower extremities). Table 1 describes characteristics of the patients and the CL lesions.

**Table 1:** Characteristics of the patients who were treated with miltefosine, n = 76.

Variable	n (%)	Contraindicated for antimonials	Failures to antimonials
Total	**76 (100)**	**42 (100)**	**34 (100)**
male	45 (59.2)	22 (52.4)	23 (67.6)
female	31 (40.8)	20 (47.6)	11 (32.4)
Age (year)			
<5	1 (1.3)	0 (0.0)	1 (2.9)
≥ 5 & < 15	20 (26.3)	0 (0.0)	20 (58.9)
≥ 15 & < 40	5 (6.6)	0 (0.0)	5 (14.7)
≥40 & < 50	13 (17.1)	9 (21.4)	4 (11.8)
≥50	37 (48.7)	33 (78.6)	4 (11.8)
Duration of lesion (months)			
1–3	8 (10.5)	4 (9.5)	4 (11.8)
4–6	23 (30.3)	12 (28.6)	11 (32.3)
7–12	36 (47.4)	22 (52.4)	14 (41.2)
>12	9 (11.8)	4 (9.5)	5 (14.7)
No. of lesions per patient			
1	40 (52.6)	21 (50.0)	19 (55.9)
2	14 (18.4)	8 (19.0)	6 (17.6)
3	9 (11.9)	3 (7.1)	6 (17.6)
4 or more	13 (17.1)	10 (23.8)	3 (8.8)
Location of the lesions			
Total of lesions	**191 (100)**	**72 (37.7)**	**119 (62.3)**
Total of face lesions	**97 (50.8)**	**46 (63.9)**	**51 (42.9)**
Cheek (s)	40 (41.2)	20 (43.5)	20 (39.2)
Nose	26 (26.8)	20 (43.5)	16 (31.4)
Forehead	14 (14.4)	7 (15.2)	7 (13.7)
Lip	6 (6.2)	2 (4.3)	4 (7.8)
Ear	5 (5.2)	2 (4.3)	3 (5.9)
Eyelid	3 (3.1)	3 (6.5)	0 (0.0)
Chin	3 (3.1)	2 (4.3)	1 (2.0)
Total upper extremities	**52 (27.2)**	**19 (26.4)**	**33 (27.7)**
arm	30 (57.7)	14 (73.7)	16 (48.5)
hand	18 (34.6)	5 (26.3)	13 (39.4)
wrist	4 (7.7)	0 (0.0)	4 (12.1)
Total lower extremities	**42 (22.0)**	**7 (9.7)**	**35 (29.4)**
leg	28 (66.7)	5 (71.4)	23 (65.7)
foot	14 (33.3)	2 (28.6)	12 (34.3)
Type of lesion*	**76 (100.0)**	**42 (100)**	**34 (100)**
nodule	15 (19.7)	0 (0.0)	15 (44.1)
plaque	43 (56.6)	27 (64.3)	16 (47.1)
ulcer	18 (23.7)	15 (35.7)	3 (8.8)

*one most prominent lesion per patient

Of the 76 patients, 42 (55.3%) started with miltefosine due to a contraindication for systemic antimonial treatment. The other 34 (44.7%) were treated because their lesions failed to cure or relapsed after prior treatment with at least two courses of antimonials.

Of the patients in the first group (contraindicated for antimonials) the median age was 53 years (IQR 50–69), whereas in the second group (failures to antimonials) the median age was 12.5 years (IQR 7–35). There was no statistically significant difference between sexes. (Table 1). In the contraindicated group, 69.0% (29/42) of the patients had a lesion on the face, whereas those with antimonial treatment failure 94.1% (32/34) had a lesion on the face.

### Outcome assessment; initial response and final cure

Twelve patients defaulted before completing treatment, and could not be assessed. We found an overall positive initial response of 90.6% (58/64) in the remaining patients who completed treatment (Table 2). In the ‘contraindicated’ group, of the 33 patients who completed treatment, 32 (97.0%) patients showed a positive initial response and one (3.0%) patient showed initial treatment failure. This patient had a large lesion on his cheek.

**Table 2:** Initial treatment response.

Reason to start miltefosine	Initial positive response to miltefosine n (%)	Initial treatment failure of miltefosine n (%)	Total n (%)
**Contraindication to antimonials**	32 (97.0%; CI 84.2–99.9)	1 (3.0%; CI 0.08–15.8)	33 (100)
**Treatment failure antimonials**	26 (83.9%; CI 66.3–94.5)	5 (16.1%; CI 5.5–33.7%)	31 (100)
**Total**	**58 (90.6%; CI 80.7–96.5)**	**6 (9.4% CI; 3.5–19.3)**	**64 (100)**

In the ‘treatment failure’ group, of the 31 patients who completed treatment, 26 (83.9%) had an initial positive response, and five (16.1%) showed an initial treatment failure. Of these, all had only one single lesion: two patients with one on the nose and three patients with one on the cheek.

Twelve patients were lost to follow up after completing treatment, and could not be assessed.

In the remaining 52 patients, the overall final cure rate was 76.9% (40/52). In the contraindicated group, the final cure rate was 82.8% (24/29), and in the failure group 69.6% (16/23) (Table 3). A non-significant difference was found in the odds of final cure between groups (OR 2.1; 95% CI 0.6–7.8) likely due to low study numbers leading to an underpowered analysis.

**Table 3:** Final treatment outcome.

Reason to start miltefosine	Final cure after miltefosine n (%)	Final treatment failure after miltefosine n (%)	Total n (%)
**Contraindication to antimonials**	24 (82.8%; CI 64.2–94.2)	5 (17.2%; CI 5.9–35.8)	29 (100)
**Treatment failure antimonials**	16 (69.6%; CI 47.1–86.8)	7 (30.4%; CI 13.2–52.9)	23 (100)
**Total**	**40 (76.9%; CI 63.2–87.5)**	**12 (23.1%; CI 12.5–36.8)**	**52 (100)**

### Patients who defaulted or were Lost-To-Follow-Up

From the cohort of 76 patients who started with miltefosine, 12 (15.8%) patients did not complete the 28 days treatment course. Six patients of those 12 stopped after two or three weeks due to adverse events (severe stomach pain, vomiting); these patients all started in the first half of 2018. Afterwards, all patients were instructed to have a meal before taking miltefosine. The other six patients started treatment but did not come for a second visit, and therefore only took medication for seven days or less, and efforts to trace these patients were not successful. Initial and final treatment outcomes of these patients are unknown (Table 4).

**Table 4:** Default and Lost-To-Follow-Up patients.

	Contraindication to antimonials n (%)	Treatment failure antimonials n (%)	Total n (%)
**Defaulted** (during treatment)	9 (69.2)	3 (27.3)	12 (50)
**Lost to follow up** (did not come for follow up)	4 (30.8)	8 (72.7)	12 (50)
**Total**	**13 (100)**	**11 (100)**	**24 (100)**

Another 12 patients (15.8%) were lost to follow up. Of these patients, the final outcomes are unknown. They were discharged from the programme after completing the treatment, but did not return for a follow up visit and could not be traced. Ten of these 12 patients had an initial positive response to the treatment and the two other patients were initial treatment failures.

### Adverse events

Of the 76 patients who started miltefosine treatment, miltefosine tolerability was recorded for 70 patients (91.1%). Of these, 42 (60%) did not experience adverse events. In the 28 patients who did suffer from AEs, a total of 39 AEs were reported (Table 5). Of these AE’s, 31 (79.5%) were mild and eight (20.5%) were moderate. There were no serious adverse events. Gastrointestinal disturbances was the main complaint (71.8%) (28/39): nausea (41.0%), stomach pain (12.8%), vomiting (12.8%) and diarrhoea (5.1%). General malaise was reported in 40.1% (9/22) of patients in the contraindicated group. One (2.6%) patient had an increase in existing eczema during treatment. The mild AEs were transient and did not require intervention or temporary discontinuation. Of the eight moderate AEs, three (37.5%) were stomach pain, two (25%) vomiting, two (25%) nausea, and one (12.5%) general malaise. In six of the eight patients with moderate AEs, these AEs were experienced as intolerable, and reason to discontinue treatment and default. These patients did not receive instruction to take the miltefosine capsule after a proper meal. The other two patients with vomiting, discontinued for two weeks and restarted miltefosine with antiemetics (ondansetron), health education and instructions to have a proper meal before taking miltefosine. Both patients completed the 28 days of treatment.

**Table 5:** Adverse events.

No. of patients	Contra indicated group n = 17	Treatment failure group n = 11	Total n = 28
	n (%)	n (%)	n (%)
Total adverse events reported	22 (56.4)	17 (43.6)	39 (100)
**Vomiting**	2 (9.0)	3 (17.6)	5 (12.8)
**Nausea**	8 (36.4)	8 (47.1)	16 (41.0)
**Stomach pain**	2 (9.0)	3 (17.6)	5 (12.8)
**Malaise**	9 (40.9)	1 (5.9)	10 (25.6)
**Diarrhoea**	1 (4.5)	1 (5.9)	2 (5.1)
**Skin problem**		1 (5.9)	1 (2.6)

## Discussion

Miltefosine is a FDA approved treatment for leishmaniasis and registered and used in Pakistan as such. Sometimes, dermatologists in Pakistan provide miltefosine to CL patients because the first line treatment with pentavalent antimonials is not available in the country. This is in accordance with WHO EMRO guidelines, although it is not (yet) included in the CL national country protocol of Pakistan [28]. Until now, evidence is lacking for efficacy of drugs other than pentavalent antimonials for the treatment of CL caused by *L*. *tropica*. However, as outlined earlier, patients with contraindications to antimonial treatment, or failure and relapse following treatment with antimonials have currently little in the way of second-line options. For this reason, MSF introduced miltefosine in its CL programme. Our observational retrospective study showed that miltefosine is an effective treatment option in CL patients with contraindications to, or previously had failed antimonial treatment. The results of this study are comparable to the outcomes of miltefosine treatment in CL caused by *L*. *major* and in muco-cutaneous leishmaniasis [22,24,25,30,31,43,44]. We showed that the overall positive initial treatment response to miltefosine was 90.6% (95%CI: 80.7–96.5%) and the overall final cure rate 76.9% (95%CI: 63.2–87.5%).

The majority (94.1%) of the patients in the antimony treatment failure group had a lesion on the face, of whom 30.4% (7/23) also failed to heal or relapsed on miltefosine. Seven of the eight patients who showed positive initial treatment response to miltefosine but failed to reach final cure were patients who had relapsed to antimonials before. Four of these seven patients were subsequently successfully treated with a combination therapy of both meglumine antimoniate (intramuscular) and miltefosine for 28 days.

In this cohort, there were more male than female patients (59.2% vs 40.8%). The reason could be the gender inequity in access to (further) CL treatment; there are more social barriers for women to seek health care. In Balochistan, women usually are required to be accompanied by a male family member when they visit a clinic. Another explanation could be that men are more affected by CL due to higher exposure to the vector, as females are usually more covered with clothes than men, even indoors (the sandfly *P*. *sergenti*, which is responsible for transmitting *L*. *tropica*, is endophilic and its preferred habitat is indoors [45]).

A limitation of the study was the low number of patients in the cohort, which did not allow for appropriate comparison between the sub-groups. In the antimony contraindication group, the failure rate was 17.2% (5/29), which is lower compared to the antimony treatment failure group with 30.4% (7/23). This difference was not significant (OR 0.48, 95% CI 0.13–1.77), however the number of subjects was too low to yield sufficient statistical power to conclude if there was a difference in cure/failure rates between the two groups.

A second limitation was that the study had a retrospective observational cohort design with data previously collected from the routine programme. These data included a relatively high number of patients who defaulted (n = 12) or were lost to follow up (n = 12). Consequently, we suffered from a high number of missing data in the outcome. Regarding the default rate, this was highest in the beginning of the programme and decreased later on. After the first defaulters, monitoring of patients and health education was intensified. The health education emphasized the importance to consume a proper meal before taking miltefosine to prevent the adverse events. This reduced the number of defaulters. It should be noted that in general CL patients in Pakistan are highly motivated to take the treatment, due to the stigma and discrimination, which can be very serious and severe in this context. Regarding the Lost to Follow up patients, it was not always possible to trace patients when they did not come for the follow up visit after completing their treatment. In the routine CL treatment programme, the patients are asked and advised to come for follow up visit, but it is not mandatory. These follow up visits in the routine CL treatment programme are to capture treatment failures and used as *proxy* for cure. For this reason, not all patients in this study came exactly on the agreed date. The patients who did come for final follow up visits came between one and three months post treatment (between Day 60 and Day 120). During the assessments on these days, the CL lesions were all screened according to the definitions for final cure and failure.

Another limitation of this study was that a parasitologically confirmed diagnosis was difficult to obtain. The sensitivity of microscopy, the gold standard used in the routine programme, varies between 70 and 90% [36,38,46–48]. There are CL lesions in which parasites cannot be found, since the yield of organisms is variable and sometimes zero, especially when the period between the first appearance of the lesion and the diagnosis is relatively long, or the CL presentation is atypical (37, 48). In this study 28.9% (22/76) of the patients with a negative CL lab result, all had a strong clinical suspicion of CL. An experienced dermatologist made the clinical diagnosis after thorough assessment and excluding differential diagnoses in these cases. In addition, molecular specification could not be done, and it was assumed that the patients were suffering from CL caused by *L*. *tropica*, based on clinical research performed at the same hospital (MSBBGHQ) in November 2017, where 100% of tested CL lesions were caused by *L*. *tropica* [27].

Tolerability was sub-optimal, with 40% of the patients suffering from adverse events, of which 71.8% were gastrointestinal disturbances and 25.6% general malaise. Due to the adverse events two patients temporarily, and six patients permanently discontinued treatment.

In conclusion, this retrospective analysis of a cohort of 76 patients showed favourable effectiveness of miltefosine as treatment for CL patients who had contraindications to systemic antimonial treatment or who had previously failed antimonial treatment. While our results are encouraging, well-powered prospective randomised controlled trials and clinical studies with systematic follow up are needed to obtain definitive evidence on the effectiveness and tolerability of miltefosine in treatment of CL caused by *L*. *tropica*, including identification of risk factors for failure to miltefosine treatment.

## Supporting information

S1 STROBE Checklist for observational retrospective studies, miltefosine in Pakistan. (DOC)
